# Dietary tea tree (*Melaleuca alternifolia*) oil supplementation enhances the expressions of amino acid transporters in goat ileal mucosa and improves intestinal immunity

**DOI:** 10.1002/fsn3.2972

**Published:** 2022-07-13

**Authors:** Xiaokang Lv, Liang Chen, Chuanshe Zhou, Yibing Guo, Guijie Zhang, Jinhe Kang, Zhiliang Tan, Shaoxun Tang, Zixin Liu

**Affiliations:** ^1^ CAS Key Laboratory for Agro‐Ecological Processes in Subtropical Region, National Engineering Laboratory for Pollution Control and Waste Utilization in Livestock and Poultry Production, Hunan Provincial Key Laboratory of Animal Nutrition Physiology and Metabolic Process Institute of Subtropical Agriculture, Chinese Academy of Sciences Changsha China; ^2^ College of Advanced Agricultural University of Chinese Academy of Sciences Beijing China; ^3^ Shenyang Agricultural University Institute of Rural Revitalization Strategy Shenyang China; ^4^ School of Agriculture Ningxia University Yinchuan China

**Keywords:** amino acid transporter, goat, intestinal immunity, tea tree oil

## Abstract

Tea tree oil (TTO) is a plant‐derived additive with anti‐inflammatory, bactericidal, and growth‐promoting properties. However, little is known about the effects of TTO on intestinal amino acid transport and immune function in goats. Twenty‐four Ganxi goats (initial body weight of 13.5 ± 0.70 kg) were randomly allotted two treatments and fed either control (CON) or CON+TTO (0.2 ml/kg) diet. The addition of TTO to the diet significantly decreased (*p* < .05) tumor necrosis factor‐α content and increased (*p* < .05) interleukin‐2 (IL‐2) content in goat serum; significantly decreased (*p* < .05) IL‐12, and increased (*p* < .05) IL‐2 content in goat ileal mucosa; significantly increased (*p* < .05) secreted IgA content in the jejunal and ileal mucosa; significantly upregulated (*p* < .05) IL‐2 and downregulated (*p* < .05) IL‐12 at the mRNA level in the ileal mucosa; significantly elevated the levels of serine, arginine, and total amino acids in the ileal mucosa (*p* < .05); significantly upregulated (*p* < .05) SLC1A1 and SLC7A1 in the ileum; and significantly enhanced (*p* < .05) the protein expression of Claudin‐1 in the ileal mucosa. In summary, adding 0.2 ml/kg of TTO to the diet enhanced SLC1A1 and SLC7A1 mRNA expression in the ileal mucosa, and SLC1A1 and SLC7A1 could transport serine and arginine from the chyme to the ileal mucosa. Thus, increased serine and arginine content in the mucosa could improve intestinal immunity. TTO supplementation upregulated the expression of IL‐2 and Claudin‐1 in goat ileal mucosa, and enhanced immune function in the intestine.

## Graphical abstract

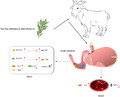

## 1 INTRODUCTION

In goat breeding, many antibiotics are used in the feed to reduce the occurrence of diseases. However, considering the residue of antibiotics in animal products and the harm to human health, China banned the use of antibiotics in animal feed in 2020 (Dong et al., 2019).

Plant essential oils are rich in natural active substances such as terpenes, esters, ketones, and alcohols (Burt, 2004). Plant essential oils have attracted the attention of animal husbandry researchers because of their antibacterial, insecticidal, and antioxidant functions (Cappelli et al., 2021; Davila‐Ramirez et al., 2020; Hall et al., 2021; Su et al., 2020; Zhang et al., 2021). Thus, plant essential oil can promote animal growth and improve intestinal immunity (Puvaca et al., 2020; Zhang et al., 2021). Nehme et al. (Nehme et al., 2021) recognized the immunomodulatory molecules of essential oils as a potential therapeutic option in ruminants and monogastric husbandries.

Tea tree oil (TTO), a plant essential oil, is distilled from the fresh branches and leaves of *Melaleuca alterniflora* and is widely used in medicine and agricultural production (Puvaca et al., 2019). TTO treatment can lead to the loss of bacterial cytoplasmic content, disrupt the integrity of the cell membrane, and ultimately lead to bacterial death (Carson et al., 2002). TTO exerts anti‐inflammatory effects by stimulating lymphocyte proliferation and inhibiting the production of proinflammatory cytokines (Brand et al., 2002). Studies have shown that adding TTO to broiler diets can significantly increase their daily weight gain (Cui et al., 2018; Khattak et al., 2014). Improvements in growth performance are often accompanied by changes in the animal body protein metabolism of animals (Xu et al., 2016). TTO supplementation can improve intestinal immunity in piglets (Dong et al., 2019). Adding Chinese herbal medicine powder to the piglet diet could increase the concentration of amino acids in the piglet's serum and promote the absorption of amino acids by the piglet's gastrointestinal tract (Kong et al., 2009). The concentration of amino acids in the blood is affected by their absorption and transport of amino acids in the gastrointestinal tract. The transport of intestinal amino acids is also closely related to muscle protein metabolism and intestinal immunity (Kong et al., 2018; Li et al., 2007). However, to the best of our knowledge, there has been no research on the effects of TTO on intestinal amino acid transport and intestinal immunity in goats. We hypothesized that the addition of TTO would improve intestinal mucosal immunity in goats. This study explored the effects of TTO on goat intestinal amino acid transport and immunity by adding TTO to goat diets.

## 2 MATERIAL AND METHODS

The study was approved by the Institutional Animal Care Committee, and all procedures involving animals were conducted following the guidelines on animal care of the Institute of Subtropical Agriculture, Chinese Academy of Sciences.

### 2.1 Animals and experimental design

Animal experiments were performed at the Institute of Subtropical Agricultural, Chinese Academy of Sciences (Changsha, China), from April to July 2019. Twenty‐four Ganxi goats (initial body weight of 13.5 ± 0.70 kg) were randomly allotted two treatments and fed either a control (CON) or CON+TTO (0.2 ml/kg) diet. The trial lasted 60 days. During the experiment, the goats had free access to feed and water. Goats were fed twice at 08:00 a.m. and 16:00 p.m. During the animal trial, all goats had access to water and feed ad libitum. The diet was formulated according to the feeding standard of meat‐producing sheep and goats (NY/T816‐2004). The formulas and nutritional levels of the diets are presented in Table 1. TTO was purchased from Wanjiahui Perfume Co. Ltd. The main component of TTO was terpinene‐4‐ol, with a content of 38.44% and a density of 0.898 g/ml.

**TABLE 1:** Composition and nutrient levels of experimental diets (DM basis) %

Ingredients	% of DM
Straw	50.0
Corn	24.4
Fat powder	8.1
Soybean meal	8.0
Soy protein concentrate	6.5
Calcium carbonate	0.7
Calcium hydrogen phosphate	0.7
Salt	0.6
Premix^a^	1.0
Total	100
Nutrition level, %	
DE (MJ/kg)^b^	10.05
CP	12.30
NDF	40.15
ADF	29.82
Ca	0.63
TP	0.31

Abbreviations: ADF, acid detergent fiber; Ca, calcium; CP, crude protein; DE, digestive energy; DM, dry matter; NDF, neutral detergent fiber; TP, total phosphorus.

^a^ The premix provided the following per kg of diets: Fe 1.5 g/kg, Cu 0.5 g/kg, Co 0.0055 g/kg, I 0.0075 g/kg, Mn 3 g/kg, Zn 2.5 g/kg, Se 0.0025 g/kg, Va 419491.5254 IU/kg, Ve 12711.86441 IU/kg.

^b^ Nutrient levels were all measured except DE.

### 2.2 Feed samples collection and analyses

Approximately 500 g of feed samples was collected and dried in an oven at 65 °C for 48 h. Feed samples were stored at −20 °C until further analysis. The determination of CP, NDF, ADF, Ca, and P in feed samples was based on the Association of Official Analytical Chemists (AOAC, 1990).

### 2.3 Determination of immunoglobulin and cytokine content in serum, jejunum, and ileal mucosa

Total serum, jejunum, and ileum mucosa protein concentrations were evaluated using the BCA Protein Assay Kit (goat#CW0014S, CWBIO). Total protein concentration was measured using a microplate reader (Infinite M200 PRO, Tecan) at 562 nm. The concentrations of interleukin‐β (IL‐1β), interleukin‐2 (IL‐2), tumor necrosis factor‐α (TNF‐α), immunoglobulin G (IgG), immunoglobulin M (IgM), and immunoglobulin A (IgA) in goat serum, and jejunal, and ileal mucosa were determined using commercial ELISA kits (goat# CSB‐E16568G, goat#CSB‐E12965G, goat# CSB‐E09811g, goat# CSB‐E12734G, and goat# CSB‐E13134G, goat# CSB‐E13135G, CUSABIO Biotech) according to the manufacturer's instructions. Likewise, the levels of interleukin‐10 (IL‐10), interleukin‐12 (IL‐12), interferon‐γ (IFN‐γ), and secretory immunoglobulin A (sIgA) in the goat jejunal and ileal mucosa were analyzed using commercial ELISA kits (goat# CSB‐E09809g, goat#CSB‐E12103G, goat#CSB‐E12966G, goat#E06S0003, CUSABIO Biotech) following the recommended procedures.

### 2.4 Amino acid profile of intestinal mucosa and muscle

After goat slaughter, the jejunal and ileal mucosa, and longissimus dorsi were immediately separated, and washed with precooled PBS (0.85% NaCl, 1.4 mM KH_2_PO_4_, 8 mM Na_2_HPO_4_, pH 7.4), and quickly put into liquid nitrogen and stored at −20°C until analysis. Amino acids in the intestinal mucosa and longissimus dorsi were determined as described elsewhere (Zhang et al., 2013). An L‐8800 automatic amino acid analyzer (Hitachi) was used to determine the hydrolyzed amino acid content of the jejunal, ileal mucosa, and longissimus dorsi.

### 2.5 Immunity markers, amino acid transporters, and barrier genes mRNA expression

After slaughter, the jejunum, ileum, and longissimus dorsi were immediately cut (5 cm), flushed with chilled PBS, and snap‐frozen in liquid nitrogen. Samples were stored at −80 °C until further analysis. RNAiso Plus (TaKaRa, Dalian, Code No. 9108/9109) was used to extract total RNA from the jejunum, ileum, and longissimus dorsi, while DNase I (Thermo Fisher Scientific) was used to eliminate genomic DNA. The purity and concentration of the total RNA were measured using a NanoDrop 2000 spectrophotometer (Thermo Scientific). Total RNA (1 μg total RNA was reverse transcribed to cDNA in a 20‐μl system using the Evo M‐MLV RT Kit (Accurate Biology11706, Changsha, China) following the manufacturer's instructions. Real‐time quantitative polymerase chain reaction (qPCR) was performed using SYBR Premix Ex Taq II (Takara) on an ABI‐7900HT qPCR system (Applied Biosystems) with β‐actin as the housekeeping gene. All primers were synthesized by Sangon Biotech. The primer sequences of target genes are shown in Table S1. The relative expression of the target gene mRNA was calculated using the 2‐ΔΔCT method (Livak & Schmittgen, 2001).

### 2.6 Immunohistochemical analysis of claudin1 in the intestinal mucosa

Immunohistochemistry was performed as previously described by Tian et al (Tian et al., 2020). Specifically, the slides were dewaxed sequentially, endogenous peroxidase was removed, antigen‐binding sites were exposed, and sections were exposed and permeabilized according to a previously described technique. Then, the sections were treated overnight at 4 °C with claudin‐1 antibodies (1:200, Abcam, #ab242370). The antigen–antibody response sites were detected for 30 min using HRP‐labeled secondary antimouse IgG (1:1000, Abcam, #ab205719), followed by staining with DAB (GeneTex). The nuclei were counterstained with hematoxylin. A fluorescein microscope (Olympus) equipped with DP2‐BSW software was used to acquire digital images at 400× magnification. ImageProline Plus 5.1 (Media Cybernetics) was used for image processing and analysis. The relative abundance of Claudin‐1 was calculated by dividing (integrated optical density) by the area.

### 2.7 Statistical analysis

All data were subjected to statistical analysis using SAS (version 9.4; SAS Inc.) with an independent‐sample Student's *t*‐test. A *p*‐value <.05 was considered a statistically significant difference. All visualizations were performed using GraphPad Prism 8 (GraphPad Software).

## 3 RESULTS

### 3.1 TTO supplementation could alter cytokine concentrations in goat serum and ileal mucosa

The addition of TTO to the diet significantly reduced (*p* < .05) the content of TNF‐α and increased (*p* < .05) IL‐2 in goat serum (Table 2). The levels of IL‐1β, IL‐4, IgG, IgM, and IgA were not affected (*p* > .05) by the addition of TTO (Table 2). As illustrated in Table 3, adding TTO to the diet significantly increased (*p* < .05) the IL‐2 content in the ileal mucosa of goats. TTO supplementation significantly decreased (*p* < .05) the IL‐12 content in the goat ileal mucosa. TTO significantly increased (*p* < .05) the sIgA content in the goat jejunal and ileal mucosa. However, the levels of TNF‐α, IL‐1β, IL‐10, and IFN‐γ in the goat jejunal and ileal mucosa were unaffected (*p* > .05) by TTO supplementation.

**TABLE 2:** Effect of TTO supplementation on serum immune index in goats

Items	Treatments^1^		SEM^2^	*p* value
	CON	CON+TTO		
TNF‐α (pg·ml^−1)^	68.72^a^	63.55^b^	7.08	.0412
IL‐1β (pg·ml^−1)^	23.13	21.44	3.04	.2073
IL‐2 (pg·ml^−1)^	38.35^b^	42.33^a^	2.04	.0241
IgG (g·L^−1^)	25.18	26.38	4.12	.7231
IgM (g·L^−1^)	0.82	0.94	0.11	.5462
IgA (g·L^−1^)	0.44	0.41	0.09	.8982

^a,b^Different superscript letters in the same variable indicate statistical differences (*p* < .05).TTO = tea tree oil.

Abbreviations: IgA, Immunoglobulin A; IgG, Immunoglobulin G; IgM, Immunoglobulin M; IL‐1β, Interleukin‐1β; IL‐2, Interleukin 2; TNF‐α, Tumor necrosis factor‐α.

^1^ CON: Control diet; CON+TTO: Control diet supplemented with 0.2 ml/kg of tea tree oil.

^2^ SEM: Standard error of the mean (overall), *n* = 12 goats per treatment.

**TABLE 3:** Effects of dietary supplementation of TTO on the contents of immunoglobulin and cytokine in goat jejunal and ileal mucosa

Items	Section	Treatments^1^		SEM^2^	*p* value
		CON	CON+TTO		
TNF‐α (pg·mg^−1^)	Jejunum	70.70	65.43	5.87	.3659
	Ileum	66.29	56.49	8.92	.5954
IL‐1β (pg·mg^−1^)	Jejunum	5.01	4.25	0.61	.5629
	Ileum	8.62	7.06	1.16	.5142
IL‐2 (pg·mg^−1^)	Jejunum	109.42	114.89	17.85	.6836
	Ileum	87.77^b^	114.63^a^	4.87	.0379
IL‐10 (pg·mg^−1^)	Jejunum	105.95	96.41	18.44	.8059
	Ileum	72.15	60.99	9.22	.5580
IL‐12 (pg·mg^−1^)	Jejunum	37.95	34.94	5.73	.8029
	Ileum	24.96^a^	15.79^b^	2.51	.0304
IFN‐γ (pg·mg^−1^)	Jejunum	149.56	148.06	16.98	.5904
	Ileum	35.73	31.64	5.22	.7057
sIgA (ug·mg^−1^)	Jejunum	0.14^b^	0.36^a^	0.05	.0187
	Ileum	0.06^b^	0.14^a^	0.01	.0145

^a,b^Different superscript letters in the same variable indicate statistical differences (*p* < .05).TTO = tea tree oil.

Abbreviations: IFN‐γ, Interferon‐γ; IL‐10, Interleukin 10; IL‐12, Interleukin 12; IL‐1β, Interleukin‐1β; IL‐2, Interleukin 2; sIgA, Secretory immunoglobulin A; TNF‐α, Tumor necrosis factor‐α.

^1^ CON: Control diet; CON+TTO: Control diet supplemented with 0.2 ml/kg of tea tree oil.

^2^ SEM: Standard error of the mean (overall), *n* = 12 goats per treatment.

### 3.2 TTO improves intestinal immunity by increasing the expression of SLC1A1, SLC7A1, and Claudin‐1 in ileal mucosa

**FIGURE 1:**
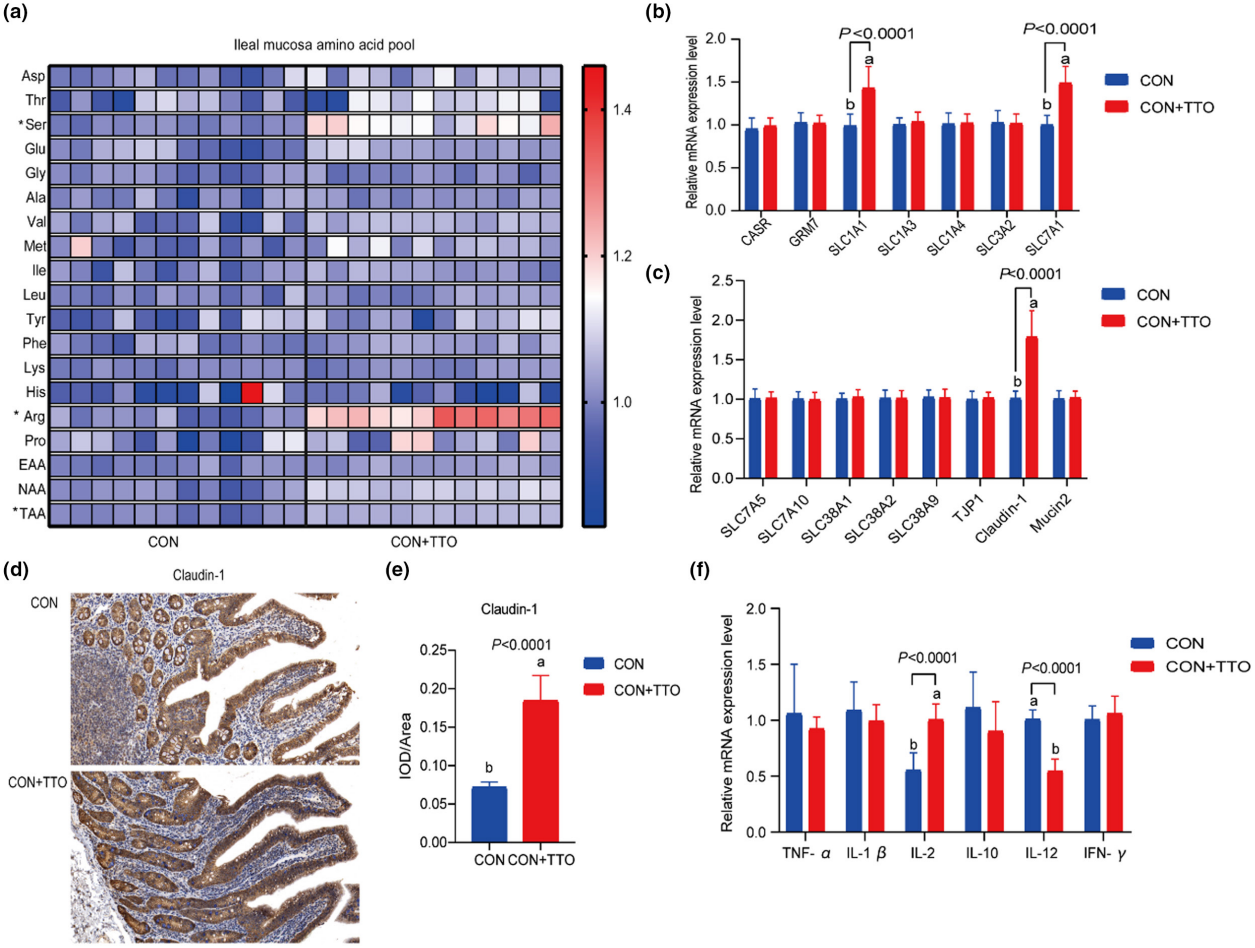
(a) Amino acid profile of goat ileal mucosa. The amino acid pool was normalized according to the mean value of the control group. * means the difference was significant compared with the control group. (b, c) the expression of mucosal barrier genes and amino acid transporters in the ileum of goats. ^a,b^ Means column with different superscripts differ (*p* < .05). (d) Expression of Claudin‐1 in ileal epithelium. (e) Comparison of average integrated optical density (IOD) of Claudin‐1 staining in ileal mucosa between CON and CON+TTO group. IOD/area: Integrated optical density per stained area. (f) the expression of immune‐related genes in the ileum of goats. TTO, tea tree oil; TNF‐α, Tumor necrosis factor‐α; IL‐1β, interleukin‐1β; IL‐2, interleukin 2; IL‐10, interleukin 10; IL‐12, interleukin 12; IFN‐γ, interferon‐γ; sIgA, secretory immunoglobulin a. CON: Control diet; CON+TTO: Control diet supplemented with 0.2 ml/kg of tea tree oil. *n* = 12 goats per treatment

As shown in Table S3, adding TTO to the diet had no significant effect (*p* > .05) on the amino acid content of the jejunal mucosa. TTO supplementation significantly increased (*p* < .05) Ser, Arg, and TAA content in the ileal mucosa (Figure 1a). The jejunal amino acid sensor receptors and transporters were not affected (*p* > .05) by TTO supplementation (Table S4). Compared to the CON group, the addition of TTO significantly enhanced (*p* < .05) the expression of SLC1A1 and SLC7A1 in the ileum (Figure 1b). TTO supplementation significantly increased (*p* < .05) the mRNA expression of Claudin‐1 in the ileal mucosa but did not affect (*p* > .05) the expression of TJP1 and Mucin2 (Figure 1c). Immunohistochemical staining showed that TTO significantly enhanced (*p* < .05) the protein expression of Claudin‐1 in the ileal mucosa (Figure 1d,e). The mRNA expression of TNF‐α, IL‐1β, IL‐2, IL‐10, IL‐12, and IFN‐γ in the goat jejunum mucosa was not affected (*p* > .05) by TTO supplementation (Figure S1). TTO supplementation significantly upregulated (*p* < .05) the mRNA expression of IL‐2 and downregulated the mRNA expression of IL‐12 in the ileal mucosa (Figure 1f).

### 3.3 Amino acid profile and the expression of genes related to protein synthesis and degradation of goat longissimus muscle

The amino acid profile of the goat muscle was not affected (*p* > .05) by TTO addition (Table 4). As shown in Table 5, there was no significant difference (*p* > .05) in the mRNA expression of muscle protein synthesis‐related genes (mTOR, 4EBP1, and S6K1) between the two groups. No significant differences (*p* > .05) were observed in the mRNA expression of FOXO3 and FOXO1 among the different treatments.

**TABLE 4:** Effect of TTO supplementation on amino acid profile in goat longissimus dorsi

Items^1^	Treatments^2^		SEM^3^	*p* value
	CON	CON+TTO		
Asp	6.75	6.53	0.08	.1452
Thr	3.23	3.44	0.07	.1096
Ser	2.38	2.35	0.04	.7221
Glu	11.37	11.19	0.24	.9122
Gly	5.48	5.38	0.14	.7350
Ala	4.81	4.91	0.10	.5960
Val	3.41	3.41	0.05	.9981
Met	1.45	1.54	0.04	.2252
Ile	3.69	3.62	0.08	.6423
Leu	6.24	6.42	0.15	.5661
Tyr	2.18	2.00	0.03	.8040
Phe	3.24	3.28	0.04	.6388
Lys	5.80	5.96	0.11	.4690
His	1.92	1.87	0.05	.5904
Arg	5.28	5.21	0.08	.6691
Pro	3.50	3.61	0.07	.4217
Flavor amino acids, FAA^c^	33.45	33.22	0.35	.7650
Essential amino acid, EAA^d^	30.20	30.53	0.22	.4929
Nonessential amino acids, NAA^e^	40.69	39.97	0.38	.8591
Total amino acids, TAA	70.89	70.50	0.41	.6572

^a,b^Different superscript letters in the same variable indicate statistical differences (*p* < .05).TTO = tea tree oil. ^c^Flavor amino acid = Glu + Asp + Ala + Arg + Gly. ^d^EAA (Essential amino acids) = Lys + Met + Thr + Val + Leu + Ile + Tyr + Phe + His. ^e^NAA (Nonessential amino acid) = Arg + His + Asp + Glu + Ala + Pro + Ser.

^1^ (DM basis, %).

^2^ CON: Control diet; CON+TTO: Control diet supplemented with 0.2 ml/kg of tea tree oil.

^3^ SEM: Standard error of the mean (overall), *n* = 12 goats per treatment.

**TABLE 5:** Effects of TTO supplementation on the expression of genes related to protein synthesis and degradation in goat muscle

Items	Treatments^1^		SEM^2^	*p* value
	CON	CON+TTO		
mTOR	1.02	1.01	0.08	.9461
4EBP1	1.06	0.94	0.09	.5996
S6K1	1.04	0.93	0.08	.5577
FOXO3	1.08	0.92	0.11	.1327
FOXO1	1.13	1.11	0.09	.2404

^a,b^Different superscript letters in the same variable indicate statistical differences (*p* < .05).

Abbreviations: 4EBPI, 4E‐binding protein 1; FOXO1, forkhead box O1; FOXO3, forkhead box O3; mTOR, mammalian target of rapamycin; S6K1, ribosomal protein S6 kinases 1; TTO, tea tree oil.

^1^ CON:Control diet; CON+TTO: Control diet supplemented with 0.2 ml/kg of tea tree oil.

^2^ SEM: Standard error of the mean (overall), *n* = 12 goats per treatment.

## 4 DISCUSSION

As a substitute for antibiotics, TTO is widely used in local alternative medicines (Carson et al., 2006; Li et al., 2016), and it has been proven to improve the antioxidant capacity of animals (Bertevello et al., 2005). However, the effect of TTO on intestinal immunity in animals remains unclear. Therefore, we used goats as experimental animals to explore the influence of TTO on intestinal immunity and the possible underlying mechanisms.

**FIGURE 2:**
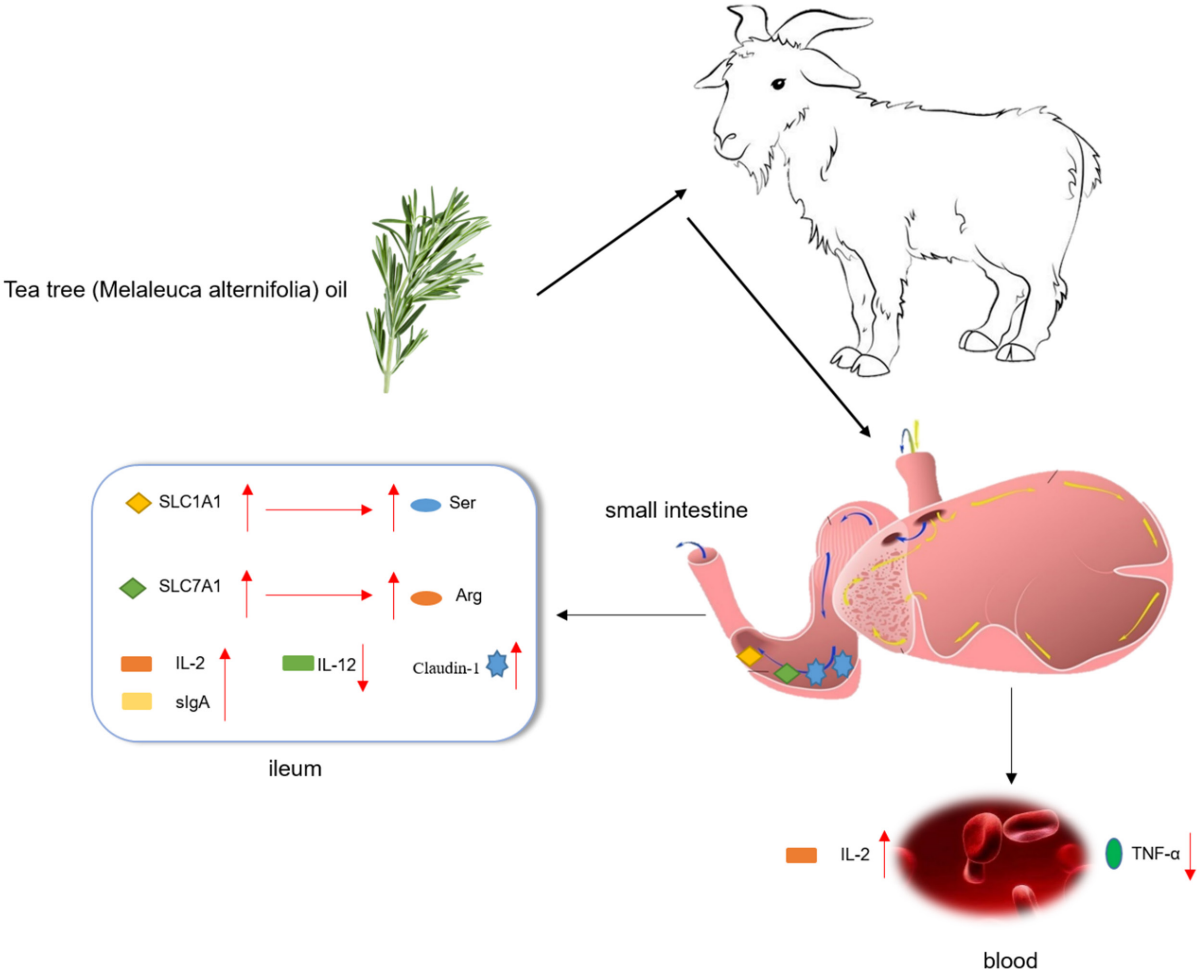
Schematic diagram for tea tree oil improving intestinal immunity by enhancing amino acid transport in the ileal mucosa of goats. Tea tree oil upregulated the expression of SLC1A1, SLC7A1 and Claudin‐1 in the ileal mucosa and increased the content of serine and arginine in the mucosa. Increased serine and arginine content elevated IL‐2 and sIgA content in the ileal mucosa and improved intestinal immunity in goats

Cytokines are considered decisive factors in the immune response of the intestinal mucosa, and can cause the production of immunoglobulins and determine the direction of T and B cell differentiation (Caldefie‐Chezet et al., 2006). In this study, we determined the concentrations of cytokines in the serum and intestinal mucosa. The results showed that the addition of TTO to the diet reduced the content of TNF‐α and increased IL‐2 in goat serum. The adding of TTO to the diet upregulated the mRNA expression of IL‐2 in the ileal mucosa. IL‐2 is an important broad‐spectrum enhancer in the body that can enhance the activity of natural killer cells, and induce T lymphocytes to produce IFN, activating immune effector cells and producing synergistic effectors, that can effectively remove tumor and virus/bacteria‐infected cells (Gaffen & Liu, 2004). Additionally, sIgA on the intestinal mucosal surface can protect the intestinal epithelium from intestinal toxins and pathogenic micro‐organisms (Geuking et al., 2012; Groschwitz & Hogan, 2009). Our results showed that TTO increased sIgA content in the goat jejunal and ileal mucosa. Under stress conditions such as trauma and infection, intestinal‐related lymphoid tissues are selectively inhibited, sIgA secretion decreases, and bacterial adhesion increases (Li et al., 2007). Our results showed that TTO can promote the secretion of sIgA in the goat intestinal mucosa to maintain intestinal health (Figure 2).

Amino acids are the building blocks of protein synthesis and act as regulatory factors that participate in the immune response (Broer, 2008). Most amino acids are metabolized in the intestine, and intestinal epithelial cells transport dietary amino acids into epithelial cells to synthesize purines, pyrimidines, and polyamines (Dan et al., 2015). Animal intestines are highly sensitive to changes in amino acids. Sufficient amino acids are essential for intestinal maintenance of mucosal integrity and immune function. Amino acids regulate the proliferation and differentiation of intestinal epithelial cells (Dan et al., 2015) and affect the intestinal epithelium's barrier function (DeMarco et al., 2003). In this study, the addition of TTO to the diet significantly increased Ser, Arg, and total amino acids in the ileal mucosa of goats. It has been reported that Ser and Arg play a critical role in maintaining intestinal integrity and regulating intestinal immune function (Qiao et al., 2005; Zhou et al., 2018; Zhu et al., 2013). Thus, dietary supplementation with Ser can prevent intestinal dysfunction (Zhou et al., 2018). Thus, dietary supplementation with Ser can prevent intestinal dysfunction. Hydroxymethyltransferase is present in the intestinal mucosa and converts Ser into Gly. N5‐N10 methyl enetetrahydrofolate is produced in this process, essential for synthesizing pyrimidine and purine (Metcalf et al., 2018). Arg participates in various nutritional and physiological processes in animals and is widely believed to promote the production of NO, proline, and polyamines in the animal body (Wu & Meininger, 2000). Therefore, the increase in Ser and Arg contents in the intestinal mucosa may be beneficial to the immune function of the intestine (Figure 2).

Amino acids cannot freely pass through the cell membrane because of their polarity, and also because they require the corresponding transport carriers to enter the cytoplasm (Verrey et al., 2005). Amino acid transporters in the mammalian intestine can be divided into anion‐amino acid transporters (SLC1A1, SLC1A2, and SLC1A3), neutral amino acid transporters (SLC1A4, SLC1A5, SLC7A5, and SLC7A10) and cationic amino acid transporters (SLC7A1, SLC7A2, and SLC7A3) (Broer, 2008). Our research showed that the amino acid transporters mRNA expression in the jejunal was not affected by TTO supplementation, which is consistent with our jejunum mucosal amino acid profile data. However, the addition of TTO to the goat diet significantly enhanced the mRNA expression of SLC1A1 and SLC7A1 in the ileum. Glu plays a vital role in enteral nutrition, cell signal transduction, and anti‐inflammatory response (Lee et al., 2007; Riazi et al., 2003; Wu, 2010). Asp and Glu are the specific substrates of SLC1A1 in the small intestine. SLC1A1 transports Asp and Glu from the intestinal lumen to the intestinal epithelial cytoplasm (Ye et al., 2016). Although our ileal mucosal amino acid profile data showed that the Asp and Glu contents in the ileal mucosa of the TTO group were only numerically higher than those of the CON group, their difference was not statistically significant. However, an increase in Glu transporter mRNA expression may improve intestinal immunity and anti‐inflammatory ability, which may be explained as an early adaptation mechanism. SLC7A1 transports Arg from the intestinal lumen to the intestinal epithelial cytoplasm. Our ileal mucosal amino acid profile data showed that TTO supplementation elevated the Arg content significantly. This suggests that TTO may transport more Arg into intestinal epithelial cells by upregulating the expression of SLC7A1 (Figure 2).

The intestinal epithelial barrier includes an intact epithelial monolayer and tight junctions (TJs). TJs are composed of occludins, claudins, tricellins, and connective adhesion molecules. Substantial evidence links impaired TJs to intestinal disease (Hering et al., 2012). Previous studies have shown that the expression of Claudin‐1 and occludin is downregulated in intestinal epithelial cells of patients with impaired intestinal absorption and defective mucosal barrier (Berkes et al., 2003; Heller et al., 2005; Kinugasa et al., 2000; Kucharzik et al., 2001). In the present study, TTO significantly increased the expression of Claudin‐1 in the ileal mucosa of goats, suggesting that tea tree oil can improve the intestinal barrier and immune function. Yong et al (Yong et al., 2022) found that TTO could enhance intestinal barrier function by increasing the expression of Claudin‐1 in the intestines of mice, which is similar to that reported in the current study (Figure 2).

The content and composition of amino acids in muscle are essential indicators for evaluating meat quality and flavor (Wood et al., 1996). Our research found that adding TTO to the diet had no significant effect on goat muscle flavor or amino acid content. This shows that the addition of TTO does not change the flavor or amino acid content of goats. Studies have claimed that TTO can improve animal growth performance (Abo Ghanima et al., 2021; Puvaca et al., 2019; Qu et al., 2019). In animals, muscle protein turnover (protein synthesis and decomposition) is a critical factor affecting growth performance, but the effect of TTO on protein turnover in muscle tissue is still unknown. The FOXO family is a transcriptional regulatory factor. FOXO1 and FOXO3 in muscle tissue are related to protein degradation (Goodman et al., 2011). Our study showed that the expression of mTOR, S6K1, 4EBP1, FOXO1, and FOXO3 in goat muscle was not affected by TTO supplementation. This indicates that tea tree oil had no adverse effect on goat muscle growth and protein metabolism.

## 5 CONCLUSIONS

In summary, adding 0.2 ml/kg of TTO to goat diets can enhance the expression of SLC1A1 and SLC7A1 in goat ileal mucosa, thereby increasing the content of Ser and Arg in the ileal mucosa, which has a beneficial effect on goat intestinal immunity. TTO upregulates IL‐2 and Claudin‐1 in the ileal mucosa to protect intestinal health.

## FUNDING INFORMATION

This work was supported by the National Natural Science Foundation of China (U20A2057, 31730092) and National Basic Research and Development Program of China (2018YFD0501903).

## CONFLICT OF INTEREST

The authors declare that no conflict of interest exist.

## ETHICAL APPROVAL

The study received the approval of the institutional Animal Care Committee, and all procedures involving animals were conducted following the guidelines on animal care of the Institute of Subtropical Agriculture, Chinese Academy of Sciences.

## Supporting information


Appendix S1

## Data Availability

Data may be provided following a request to the corresponding author.
