# Supplementary material for: Dietary tea tree (Melaleuca alternifolia) oil supplementation enhances the expressions of amino acid transporters in goat ileal mucosa and improves intestinal immunity
Source: Food Sci Nutr. 2022 Jul 13;10(11):3749–58. doi: 10.1002/fsn3.2972 (PMC9632209; doi:10.1002/fsn3.2972)
Supplement: Supplementary file 1 — Appendix S1 [file FSN3-10-3749-s001.docx]

Table S1 Primer sequence of target genes

| Gene | Primer sequence (5’-3’) | | Size (bp) | References |
| --- | --- | --- | --- | --- |
| TNF-α | Forward | CCACTGACGGGCTTTACCT | 141 | ^(Chen et al., 2019)^ |
|  | Reverse | TGATGGCAGAGAGGATGTTG |  |  |
| IL-1β | Forward | AAGCCTCTCCACCTCCTCTC | 114 |  |
|  | Reverse | TTGTCCCTGATACCCAAGG |  |  |
| IL-2 | Forward | GTGAAGTCATTGCTGCTGGA | 113 |  |
|  | Reverse | GCGTTAACCTTGGGCATGTA |  |  |
| IL-10 | Forward | GCTGTTGCCTGGTCTTCCT | 178 |  |
|  | Reverse | TGTTCAGTTGGTCCTTCATTTG |  |  |
| IL-12 | Forward | ATTGAGGTCGTGATGGAAGC | 190 | This study |
|  | Reverse | GGGAGAAGTAGGAATGTGGG |  |  |
| IFN-γ | Forward | AGATCCAGCGCAAAGCCATA | 110 |  |
|  | Reverse | TCTCCGGCCTCGAAAGAGAT |  |  |
| GRM7 | Forward | ACACTGCGTCAATCACAAACTG | 156 | ^(Wu et al., 2020)^ |
|  | Reverse | TTGCCGAATACTGGGAGGA |  |  |
| CASR | Forward | CTCTTCCCTGATTGCTATGCC | 119 |  |
|  | Reverse | TCCTGGGGTGGACTTTCTG |  |  |
| SLC1A1 | Forward | AGCGGAAAGTGACAGGCAGT | 155 |  |
|  | Reverse | TGGGCTTGCAATCCACTCCA |  |  |
| SLC1A3 | Forward | ATGGGACCGCCCTCTATGA | 105 |  |
|  | Reverse | CCGTGGCTGTGATGCTGAT |  |  |
| SLC1A4 | Forward | ATCTGGCTCGCAGACCCTTC | 150 |  |
|  | Reverse | GCATACGTCCGGAAAGCTGC |  |  |
| SLC7A5 | Forward | ACGGCTGCTGACGCCTGTAC | 123 |  |
|  | Reverse | GCCACGCAGAGCCAGTTGAAG |  |  |
| SLC7A10 | Forward | CGCCTTTGACTTCTGGATGA | 143 |  |
|  | Reverse | TGGCACGAGGTAGGTTCTTT |  |  |
| SLC38A1 | Forward | GTACGGCTGGCAGTCATTGT | 127 |  |
|  | Reverse | AGGTCACCGAGACATGACGA |  |  |
| SLC38A2 | Forward | AAAGCCATTATGCCGATGTAG | 166 |  |
|  | Reverse | GAAAGCCCAAGGATTCCACT |  |  |
| SLC7A1 | Forward | ATGAAGCAGATGAGCAGGGAC | 104 |  |
|  | Reverse | GCCTTCGTGGGCTTTGACT |  |  |
| SLC3A2 | Forward | CCTCGTCGTCGGCAACCTTG | 108 |  |
|  | Reverse | CCGAGAAGCAGCCGATGAACG |  |  |
| SLC38A9 | Forward | ATTTCCTTCACCACCAT | 230 | ^(Lv et al., 2021)^ |
|  | Reverse | CTCCCACAATAAACAGAT |  |  |
| mTOR | Forward | CCTGTTCGTGGCTCTGAATGACC | 161 |  |
|  | Reverse | TTCTTCCAATGCCGCTGTGCTC |  |  |
| 4EBP1 | Forward | CTCACCTGTGACCAAGACGC | 101 |  |
|  | Reverse | GGTGATTCTGGCTGGCTTCC |  |  |
| S6K1 | Forward | CGCAGACGCCTGTGGAATACC | 95 |  |
|  | Reverse | TCGTATTGGAAGCGGTGCTGAAG |  |  |
| TJP1 | Forward | ACCACACTGTGATCCTAAAACCT | 77 | This study |
|  | Reverse | CACAGTTTGCGCCAACAAGA |  |  |
| occludin | Forward | ATCGGAGTTTCAGGTGAATGGG | 97 |  |
|  | Reverse | TCCGCCTGAAGAAGCAGAAAG |  |  |
| claudin-1 | Forward | CAGGCCTTCTCGTGGTTAGG | 93 |  |
|  | Reverse | ATGGAACAGGGTGCCAACAA |  |  |
| mucin-2 | Forward | GGACTCGCACTCATGTGGAA | 158 |  |
|  | Reverse | CCAAACTCCACGGGACTGAA |  |  |
| FOXO3 | Forward | CCAGACAAACGGCTCACTCT | 171 |  |
|  | Reverse | CTTGCCGGTTCCCTCATTCT |  |  |
| FOXO1 | Forward | AGTGGATGGTCAAGAGCGTG | 155 |  |
|  | Reverse | GAGCATCCACCAGGAGCTTT |  |  |
| β-ACTIN | Forward | CTTCCAGCCTTCCTTCCTG | 111 |  |
|  | Reverse | ACCGTGTTGGCGTAAAGGT |  |  |

Table S2. Effect of adding TTO to the diet on the growth performance of goats

| Item | Treatments^2^ | | SEM^3^ | *P* value |
| --- | --- | --- | --- | --- |
|  | CON | CON+TTO |  |  |
| ADFI^1^（g/d） | 371.08 | 343.99 | 17.69 | 0.1403 |
| ADG^1^（g/d） | 22.51 | 31.53 | 5.75 | 0.3121 |
| F/G^1^ | 24.30 | 14.55 | 5.94 | 0.1214 |

^1^ADFI: average daily feed intake; ADG: average daily gain;F/G: feed intake/ average daily gain.

^2^CON：Control diet; CON+TTO: Control diet supplemented with 0.2 ml/kg of tea tree oil

^3^SEM: Standard error of the mean (overall), n = 12 goats per treatment

Table S3 Effect of TTO supplementation on the amino acid profile of jejunum mucosa

| Items | Treatments^1^ | |  | |
| --- | --- | --- | --- | --- |
|  | CON | CON+TTO | SEM^2^ | *P* value |
| Asp | 1.34 | 1.38 | 0.05 | 0.7607 |
| Thr | 0.55 | 0.54 | 0.02 | 0.8674 |
| Ser | 0.55 | 0.58 | 0.02 | 0.3646 |
| Glu | 1.59 | 1.64 | 0.07 | 0.7135 |
| Gly | 1.86 | 1.80 | 0.05 | 0.6430 |
| Ala | 0.95 | 0.88 | 0.03 | 0.3385 |
| Val | 0.68 | 0.70 | 0.02 | 0.6188 |
| Met | 0.25 | 0.24 | 0.01 | 0.6535 |
| Ile | 0.55 | 0.54 | 0.02 | 0.7552 |
| Leu | 0.98 | 0.97 | 0.02 | 0.9004 |
| Tyr | 0.45 | 0.44 | 0.01 | 0.4814 |
| Phe | 0.62 | 0.61 | 0.01 | 0.5770 |
| Lys | 1.00 | 0.99 | 0.01 | 0.7319 |
| His | 0.30 | 0.31 | 0.01 | 0.8974 |
| Arg | 1.14 | 0.97 | 0.10 | 0.1380 |
| Pro | 0.71 | 0.70 | 0.02 | 0.7097 |
| EAA^a^ | 5.38 | 5.50 | 0.04 | 0.1216 |
| NAA^b^ | 7.97 | 7.94 | 0.09 | 0.8945 |
| TAA | 13.35 | 13.44 | 0.09 | 0.6181 |

^1^CON：Control diet; CON+TTO: Control diet supplemented with 0.2 ml/kg of tea tree oil

^2^SEM: Standard error of the mean (overall), n = 12 goats per treatment

^a^EAA = Lys + Met + Thr + Val + Leu + Ile + Tyr + Phe + His. ^b^NAA = Arg + His + Asp + Glu + Ala + Pro + Ser.

Table S4 Effect of TTO supplementation on the expression of amino acid transporter in goat jejunum mucosa

| Items | Treatments^1^ | |  | |
| --- | --- | --- | --- | --- |
|  | CON | CON+TTO | SEM^2^ | *P* value |
| CASR | 0.95 | 1.03 | 0.05 | 0.3701 |
| GRM7 | 1.03 | 0.98 | 0.09 | 0.7652 |
| SLC1A1 | 1.02 | 1.08 | 0.10 | 0.9322 |
| SLC1A3 | 1.01 | 1.05 | 0.13 | 0.1913 |
| SLC1A4 | 0.95 | 1.08 | 0.11 | 0.3078 |
| SLC3A2 | 0.99 | 1.03 | 0.02 | 0.3066 |
| SLC7A1 | 1.21 | 1.09 | 0.12 | 0.4326 |
| SLC7A5 | 1.03 | 0.97 | 0.11 | 0.9036 |
| SLC7A10 | 0.98 | 0.93 | 0.03 | 0.3841 |
| SLC38A1 | 0.99 | 1.08 | 0.11 | 0.3815 |
| SLC38A2 | 1.04 | 1.08 | 0.10 | 0.2334 |
| SLC38A9 | 0.99 | 1.05 | 0.10 | 0.3529 |

^1^CON：Control diet; CON+TTO: Control diet supplemented with 0.2 ml/kg of tea tree oil

^2^SEM: Standard error of the mean (overall), n = 12 goats per treatment

^
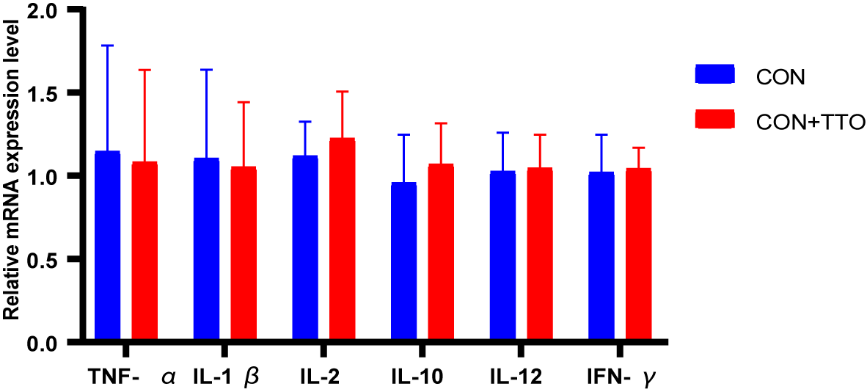
^

Figure S1. The expression of immune-related genes in the jejunum of goats. TTO = tea tree oil. Tumor necrosis factor-α: TNF-α; Interleukin-1β: IL-1β; Interleukin 2: IL-2; Interleukin 10: IL-10; Interleukin 12: IL-12; Interferon-γ: IFN-γ; Secretory immunoglobulin A: sIgA. CON: Control diet; CON+TTO: Control diet supplemented with 0.2 ml/kg of tea tree oil. n = 12 goats per treatment.

**References**

Chen, W., Q. Yan, H. Yang, X. Zhou, and Z. Tan. 2019. Effects of restrictions on maternal feed intake on the immune indexes of umbilical cord blood and liver Toll-like receptor signaling pathways in fetal goats during pregnancy. Journal of Animal Science and Biotechnology 10(3):736-744.

Lv, X., C. Zhou, T. Ran, J. Jiao, Y. Liu, Z. Tan, S. Tang, J. Kang, J. Xie, L. Chen, A. Ren, Q. Xv, and Z. Kong. 2021. Dietary amylose:amylopectin ratio influences the expression of amino acid transporters and enzyme activities for amino acid metabolism in the gastrointestinal tract of goats. The British journal of nutrition:1-11.

Wu, J., X. L. Zhang, R. Wang, M. Wang, Z. X. He, Z. L. Tan, and J. Z. Jiao. 2020. Replacing corn grain with corn gluten feed: Effects on the rumen microbial protein synthesis, functional bacterial groups and epithelial amino acid chemosensing in growing goats. Anim Feed Sci Tech 270.
